# Genetic Characterization of H1N1 and H1N2 Influenza A Viruses Circulating in Ontario Pigs in 2012

**DOI:** 10.1371/journal.pone.0127840

**Published:** 2015-06-01

**Authors:** Helena Grgić, Marcio Costa, Robert M. Friendship, Susy Carman, Éva Nagy, Zvonimir Poljak

**Affiliations:** 1 Department of Population Medicine, Ontario Veterinary College, University of Guelph, Guelph, Ontario, N1G 2W1, Canada; 2 Department of Pathobiology, Ontario Veterinary College, University of Guelph, Guelph, Ontario, N1G 2W1, Canada; 3 Animal Health Laboratory, Laboratory Services Division, University of Guelph, Guelph, Ontario, N1H 6R8, Canada; 4 Centre for Public Health and Zoonoses, University of Guelph, Guelph, Ontario, Canada; Centre of Influenza Research, The University of Hong Kong, HONG KONG

## Abstract

The objective of this study was to characterize H1N1 and H1N2 influenza A virus isolates detected during outbreaks of respiratory disease in pig herds in Ontario (Canada) in 2012. Six influenza viruses were included in analysis using full genome sequencing based on the 454 platform. In five H1N1 isolates, all eight segments were genetically related to 2009 pandemic virus (A(H1N1)pdm09). One H1N2 isolate had hemagglutinin (HA), polymerase A (PA) and non-structural (NS) genes closely related to A(H1N1)pdm09, and neuraminidase (NA), matrix (M), polymerase B1 (PB1), polymerase B2 (PB2), and nucleoprotein (NP) genes originating from a triple-reassortant H3N2 virus (tr H3N2). The HA gene of five Ontario H1 isolates exhibited high identity of 99% with the human A(H1N1)pdm09 [A/Mexico/InDRE4487/09] from Mexico, while one Ontario H1N1 isolate had only 96.9% identity with this Mexican virus. Each of the five Ontario H1N1 viruses had between one and four amino acid (aa) changes within five antigenic sites, while one Ontario H1N2 virus had two aa changes within two antigenic sites. Such aa changes in antigenic sites could have an effect on antibody recognition and ultimately have implications for immunization practices. According to aa sequence analysis of the M2 protein, Ontario H1N1 and H1N2 viruses can be expected to offer resistance to adamantane derivatives, but not to neuraminidase inhibitors.

## Introduction

Influenza A viruses are highly infectious pathogens in a wide range of mammalian and avian species. They are divided into subtypes based on two surface glycoproteins, hemagglutinin (HA) and neuraminidase (NA). Eighteen HA and eleven NA subtypes have been identified so far [1].

Both H17N10 and H18N11 subtypes have been detected in bats, while other subtypes were found in waterfowl and shore birds [2,3].

The first clinical report of influenza-like disease in pigs originated in United States during the pandemic of 1918–1919 [4]. The early viruses classified as classical swine H1N1 were genetically and antigenically similar to the type A influenza viruses acquired from humans during the 1918 influenza pandemic [5]. For almost 80 years, classical swine H1N1 was stable and was the only predominant subtype in the North American swine population, although Chambers et al. [6] showed that human subtype H3 influenza viruses were circulating among U.S. pigs at a low frequency. In 1998, Zhou et al. [7] reported double- and triple-reassortant (tr) H3N2 viruses capable of causing disease in pigs. The main difference between those two H3N2 groups was the acquisition of avian polymerase A (PA) and polymerase B2 (PB2) genes in the trH3N2. Almost all North American influenza A virus in swine (IAV-S) reassortants contain an internal gene combination composed of the PA and PB2 genes of avian lineage, NS, NP, and M genes of classical swine lineage, and the polymerase B1 (PB1) gene of human lineage. This triple-ressortant internal gene (TRIG) cassette seems to have a high potential for accepting multiple HA and NA types, resulting in increased detection of new reassortment influenza viruses. In 2009, an influenza pandemic started in the human population, caused by a novel reassortant H1N1 virus which contained neuraminidase (NA) and matrix (M) gene segments derived from the Eurasian lineage of swine IAV. The rest of the genes were from North American tr IAV-S [8,9]. According to phylogenetic analysis, A(H1N1)pdm09 originated from swine, and once in humans, the virus was transmitted back to the swine population and continued to reassort with other IAV-S [10,11]. The phylogenetic studies of Larusso et al. [12] and Vincent et al. [13] led to grouping of the IAV-S H1 subtype into α, β, γ and δ clusters.

One of the areas of improvement for studying influenza is increased surveillance of influenza viruses in swine [8]. Virological surveillance of influenza is important from multiple perspectives, including evaluation of trends for animal and public health, and providing information for potential vaccine updates. Earlier studies have described the circulation of several unique viruses in Ontario swine, in addition to classical swine influenza H1N1 virus. In 1997 a wholly human H3N2 virus was isolated from a baby pig with respiratory disease and was genetically characterized [14]. A fully avian H4N6 was isolated in the fall of 1999 from a commercial swine herd that drank surface lake water, and was also subsequently genetically characterized [15]. Wholly avian H3N3 viruses were isolated from the same Ontario swine herd in the fall of 2001 [16]. A completely human H1N2 was isolated in 2003 and 2004, with subsequent human-swine H1N2 reassortants isolated in 2003 and 2004 and genetically characterized [17]. In addition a unique human-swine reassortant H1N1, containing a human lineage PB1 polymerase gene, with all other genes of classical H1N1 swine influenza A, was isolated in 2003 and 2004 [17]. In 2005 a triple-reassortant human-avian-swine H3N2 virus was isolated from Ontario swine [18]. Subsequently, surveillance of influenza viruses in Ontario swine continued as summaries of influenza A virus H1N1 and H3N2 isolations from 1998 to April 2009, and were reported by the Animal Health Laboratory [19]. However, these summary reports did not include detailed molecular characterization. Ontario is the province with the largest number of pig farms and with the second largest number of pigs on farms in Canada [20]. It is important that molecular characterization of Ontario swine influenza viruses be continued. Therefore, the objective of this study was to isolate and genetically characterize H1N1 and H1N2 isolates detected during outbreaks of respiratory disease in pig herds in Ontario (Canada) in 2012.

## Material and Methods

### Viruses and virus isolation

Viruses in this study were isolated from Ontario pigs in 2012 as described by Grgic et al. [21]. Briefly, Dacron-tipped nasal swabs were deposited in 2 ml of phosphate buffered saline (PBS) containing 100 U/mL of penicillin and 100 μg/mL of streptomycin, and transported to the virology laboratory (Department of Pathobiology, University of Guelph). Animal Care Committee of the University of Guelph approved the study, AUP#1277. Samples were inoculated into Madin-Darby canine kidney (MDCK) cells as described elsewhere [22]. Culture supernatants were tested for the presence of IAV-S by hemagglutination (HA) assay using chicken red blood cells, as described by the WHO manual on animal influenza diagnosis and surveillance [22].

### RNA extraction and RT-PCR

RNA extraction and real-time reverse transcription PCR (rtRT-PCR) were carried out as described by Grgic et al. [21]. Briefly, viral RNA was extracted on an automated platform using the MagMAX Express-96 instrument and MagMAX 96 Viral RNA Isolation kit (cat#AM1836), according to the manufacturer’s instructions. IAV-S was identified by rtRT-PCR with matrix primers redesigned by Dr. J. Pasik (CFIA, Winnipeg, MB, CA) enhanced for the detection of A(H1N1)pdm09 [21]. The subtype was determined by rtRT-PCR and further characterized by full genome sequencing utilizing 454 sequence technology as described by Grgic et al. [21]. Five isolates typed as H1N1 and one isolate typed as H1N2 were included in the full genome sequencing study.

### Genomic sequencing and sequence assembly

The complete nucleotide sequences of 6 viruses were determined using the 454 GS Junior Titanium platform (Roche Applied Science, Indianapolis, IN, USA) as described by Grgic et al. [21]. Briefly, RNA of all 6 Ontario 2012 viruses was fragmented by ZnCl_2_ into fragments between 500 bp and 1500 bp. Sheared RNA was used for first and second strand cDNA synthesis and for fragment end repair. Double-stranded cDNA purification was performed using AMPure beads and Magnetic Particle Concentrator (MPC). Once the End Repair program was completed the 454 rapid library multiplex identifier (RL MID) was ligated to the fragments according to the GS Junior Titanium cDNA rapid library preparation method (Roche). Quality assessment of the RNA samples was performed using the FlashGel system. Sequence assembly was done with the Newbler (version 2.5p1) *de novo sequence* assembly software (Roche).

### Sequence analysis

To determine gene relatedness of each gene segment of the Ontario swine H1N1 isolates, we used BLAST from the GenBank database. Geneious Pro 5.5.6. has been used to determine nucleotide and aa sequence identities. The CLUSTAL W alignment method was applied, and an unrooted phylogenic tree of the HA, NA, and M genes was constructed by using Jukes-Cantor genetic distance model and UPGMA tree-building method.

### Crystal structure manipulations

The aa sequences of the HA1 subunit of all 6 Ontario H1 isolates were aligned with those of a human isolate from Mexico [A/Mexico/InDRE4487/09] to identify aa substitutions with the five antigenic sites Sa, Sb, Ca1, Ca2 and Cb. The HA crystal structure of the H1 subtype influenza virus [A/California/04/2009(H1N1)] (PDB 3AL4.A) was used as reference. Molecular graphics images were produced using PyMOL (http://www.pymol.org). Resulting images were imported into GIMPShop and assembled.

## Results

### Genetic characterization of 5 Ontario H1N1 and 1 H1N2 influenza virus segments

All 8 segments from 5 Ontario H1N1 and 1 Ontario H1N2 isolates have been sequenced and the sequences deposited in the GenBank database under accession numbers KM489563-KM489610. The sequences generated from this study were compared to the H1N1 viruses isolated from pigs in Canada during 2009 as described by Nfon et al. [23], H3N2 viruses isolated from Ontario during 2011–2012 [21], A(H1N1)pdm09 viruses, and other IAV published in GenBank.

All 5 Ontario H1N1 viruses had all segments originating from the 2009 A(H1N1)pdm09 virus. The Ontario H1N2 virus had HA, PA and NS genes originating from A(H1N1)pdm09 virus, and NA, M, PB1, PB2, and NP originating from the trH3N2 virus.

**Fig 1:**
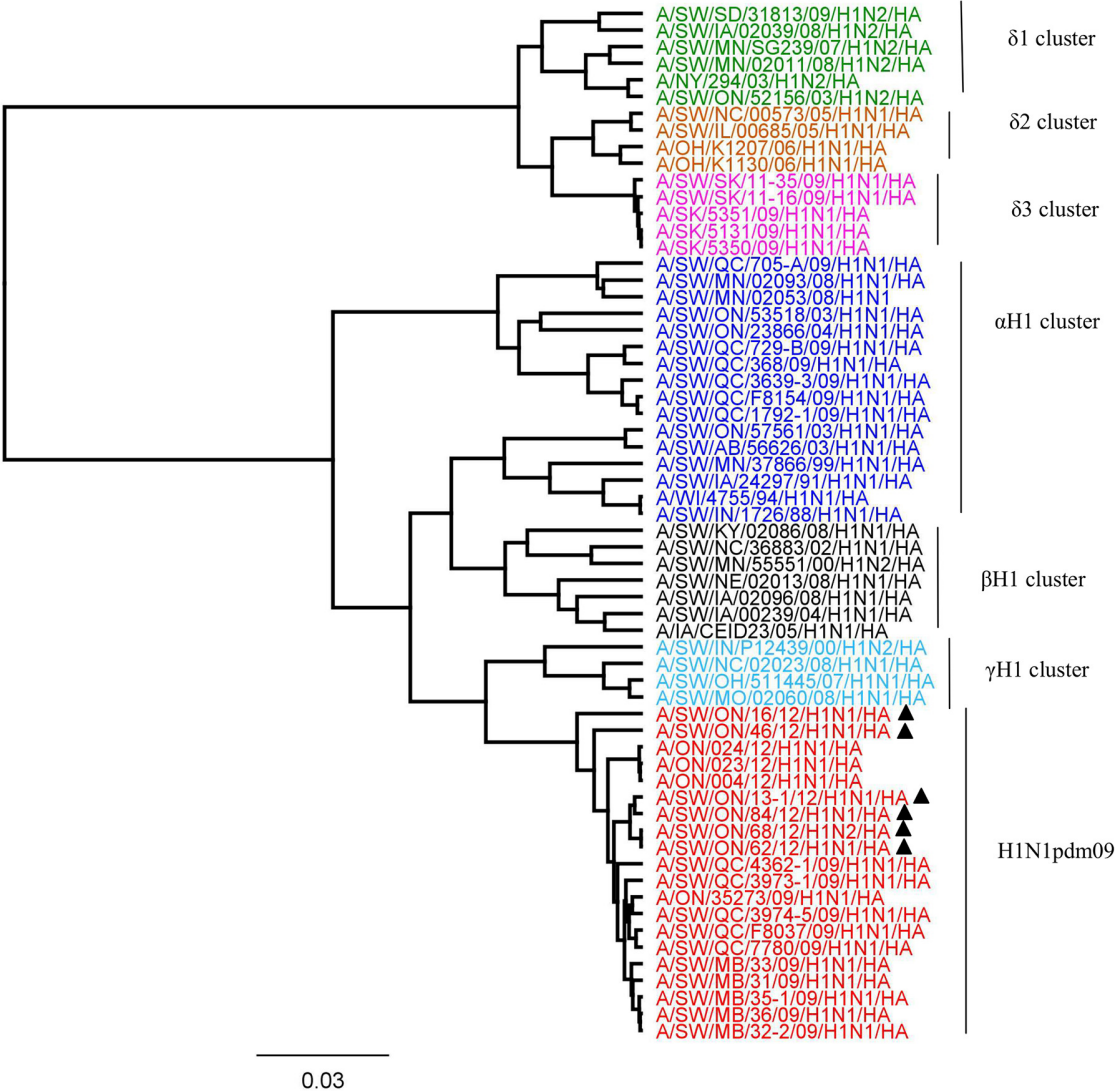
Phylogenetic analysis of the hemagglutinin (HA) gene of six Ontario H1 subtype viruses isolated from pigs in 2012(▲). Influenza A virus in swine (IAV-S) H1 α (blue), β (black), γ (turquoise), δ1 (green), δ2 (brown), δ3 (pink) clusters and pandemic (H1N1pdm09) (red) Canadian H1N1 viruses have been included in analysis.

**Fig 2:**
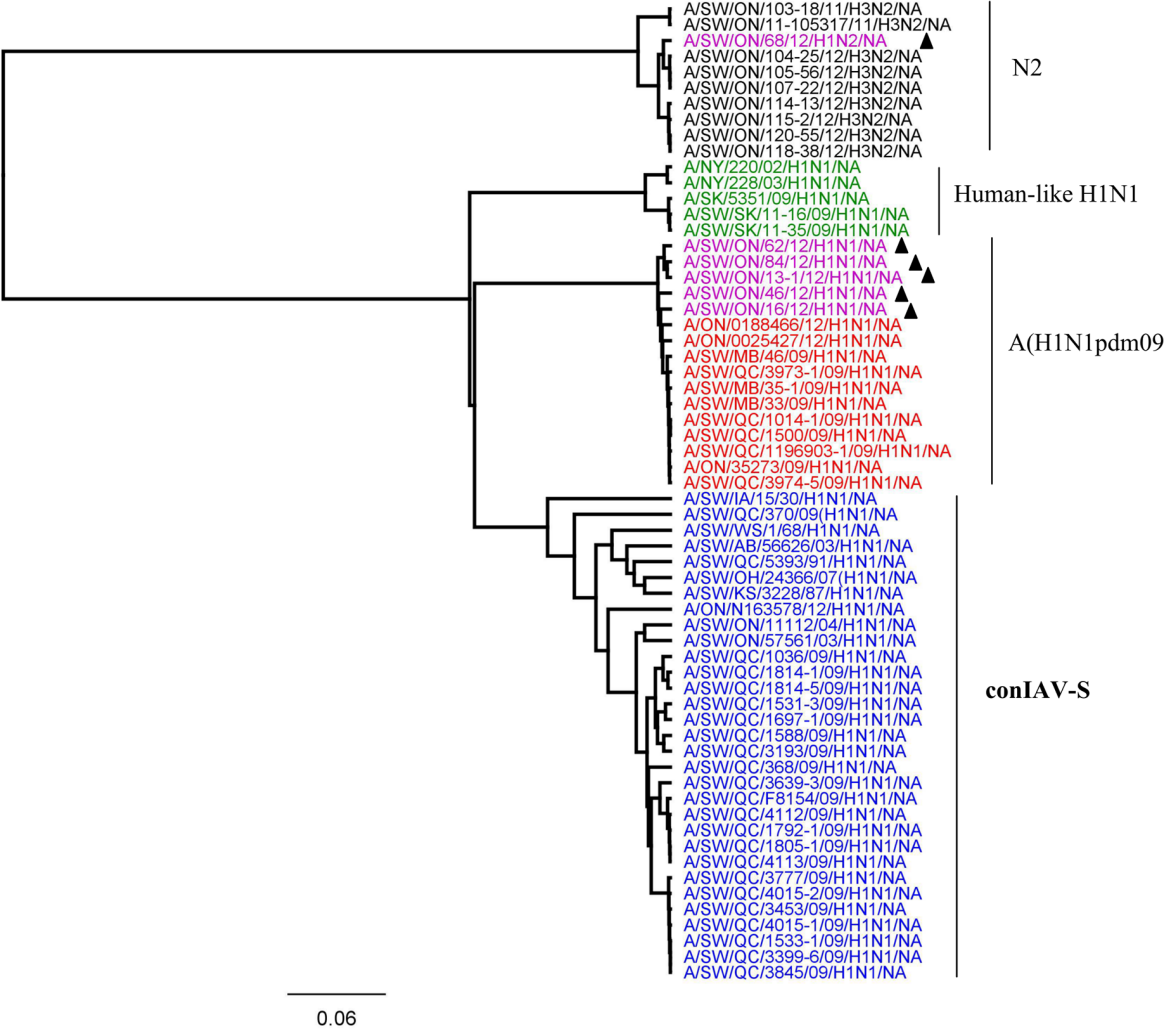
Phylogenetic analysis of neuraminidase (NA) gene of 5 Ontario H1N1 and 1 H1N2 viruses isolated from pigs in 2012 (purple with ▲). The H3N2 viruses isolated from Ontario swine between 2011 and 2012 (black), human-like H1N1 bearing seasonal human H1 and N1 genes together with the triple-reassortant internal gene (TRIG) cassette (green), A(H1N1)pdm09 (red), and contemporary influenza A viruses from swine (conIAV-S) which contain HA and NA genes of classical H1N1 in combination with the North American TRIG cassette (blue) have been included in analysis.

**Fig 3:**
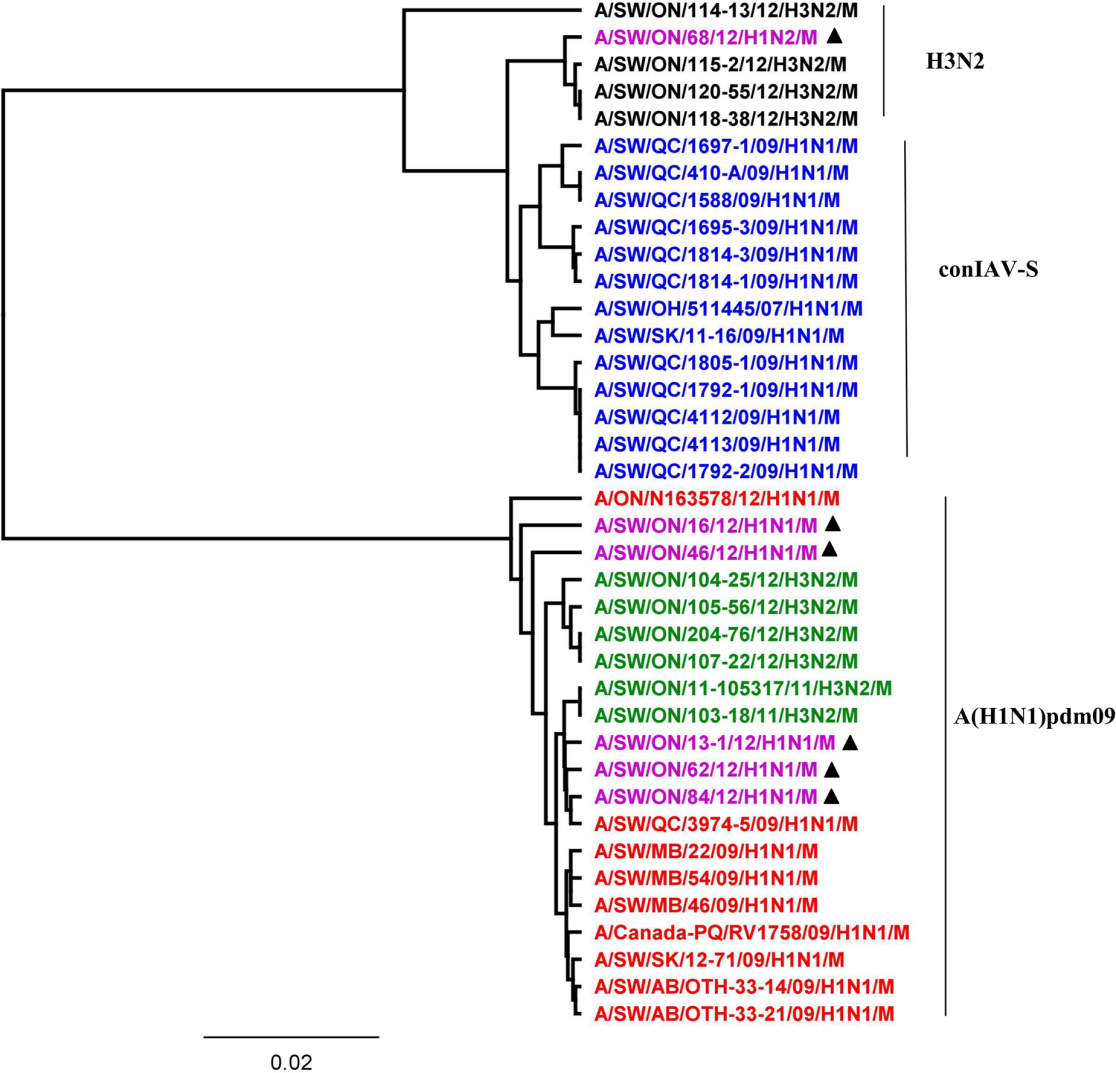
Phylogenetic analysis of the matrix (M) gene of 5 Ontario H1N1 and 1 H1N2 viruses isolated from pigs in 2012 (purple with ▲). The H3N2 viruses isolated from Ontario swine between 2011 and 2012 containing M gene from trH3N2 (black), contemporary influenza A viruses from swine (conIAV-S) which contain HA and NA genes of classical H1N1 in combination with the North American triple-reassortant internal gene (TRIG) cassette (blue), A(H1N1)pdm09 (red), and H3N2 viruses (green) isolated from Ontario swine between 2011 and 2012 containing M gene from pandemic H1N1 (A(H1N1)pdm09) have been included in analysis.

Based on the HA (Fig 1), NA (Fig 2), and M (Fig 3) genes, the 2012 H1N1 viruses isolated from Ontario pigs were clearly distinct from the classical swine H1N1 viruses (Figs 1, 2, and 3). The HA, NA, and M gene sequences of Ontario H1N1 viruses clustered with previously described A(H1N1)pdm09 viruses isolated from swine and humans. More detailed analysis of HA, NA, and M genes showed that the 5 Ontario H1N1 isolates were indeed most similar to the A(H1N1)pdm09 from 2009 with 97.6% to 99.1%, 98.3% to 99.1%, and 98.6% to 99.5% identity of HA, NA, and M genes, respectively (data not shown).

The M pairwise nucleotide identities between the 5 Ontario H1N1 viruses and their counterparts of Ontario H3N2 viruses containing an M gene genetically related to A(H1N1)pdm09 [21] varied between 98.4% to 99.7%. Overall, nucleotide sequence identities between the HA, NA, and M genes of the 5 Ontario H1N1 isolates analyzed in this study ranged from 96.3% to 99.7% for HA, 97.8% to 99.6% for NA, and 98.2% to 99.7% for M gene segment (data not shown for percent identities).

The remaining internal genes PB1, PB2, NP, NS, and PA were also genetically related to A(H1N1)pdm09. Their nucleotide sequence identities were compared to their respective A(H1N1)pdm09 [A/Netherlands/602/09/] and [A/ON/9739/09] counterparts, and varied between 98.6% and 99.6%, 98.6% and 99.1%, 98.1% and 99.2%, 98.3% and 99.3%, 98.6% and 99.3% for PB1, PB2, NP, NS and PA genes, respectively. Overall, nucleotide sequence identities between the internal genes of the 2012 Ontario H1N1 viruses ranged from 98.3% to 99.9% for PB1, 97.9% to 99.7% for PB2, 97.8% to 99.9% for NP, 97.4% to 99.9% to NS, and 98.3% to 99.8% for PA. The percentage of genetic relatedness of the 5 Ontario H1N1 viruses to other published influenza viruses in the NCBI database are presented in Table 1. Based on the top matches for each gene sequence 5 Ontario H1N1 viruses indeed were most similar the A(H1N1)pdm09 with 97% to 100% identity.

**Table 1:** Top BLAST matches of the 8 gene segments from 5 Ontario H1N1 and 1 Ontario H1N2 virus obtained from NCBI influenza A virus nucleotides and amino acid database.

**gene**		**A/SW/ON/16/12/H1N1**	**A/SW/ON/13-1/12/H1N1**	**A/SW/ON/84/12/H1N1**
**PB2**	**NA** ^a^	99% A/Wisconsin/629D00734/2009(H1N1)	99% A/Ontario/29801/2009(H1N1)	99% A/Ontario/29801/2009(H1N1)
	**AA** ^b^	100% A/New York/4844/2009(H1N1)	99% A/Mexico City/015/2009(H1N1)	99% A/Mexico City/015/2009(H1N1)
**PB1**	**NA**	99% A/Canada-ON/RV2967/2009(H1N1)	99% A/Ontario/29801/2009(H1N1)	99% A/SW/SK/59-6/2009(H1N1)
	**AA**	99% A/Boston/DOA14/2011(H1N1)	99% A/Canada-ON/RV2964/2009(H1N1)	99% A/Canada-ON/RV2964/2009(H1N1)
**PA**	**NA**	99% A/Managua/5258.02/2009(H1N1)	99% A/Ontario/29801/2009(H1N1)	99% A/Ontario/29801/2009(H1N1)
	**AA**	99% A/Sichuan/1/2009(H1N1)	99% A/Terrassa/INS174/2009(H1N1)	99% A/Terrassa/INS174/2009(H1N1)
**HA**	**NA**	98% A/Tottori/ST338/2009(H1N1)	99% A/California/VRDL30/2009(H1N1)	99% A/California/VRDL30/2009(H1N1)
	**AA**	99% A/New York/6775/2009(H1N1)	99% A/Taiwan/99902/2009(H1N1)	99% A/Kuwait/N13013/2009(H1N1)
**NP**	**NA**	99% A/Athens/INS273/2009(H1N1)	99% A/Mexico City/INERPHAC1/2009(H1N1)	99% A/Mexico City/INERPHAC1/2009(H1N1)
	**AA**	99% A/sw/AR/SAGiles-31215/2009(H1N1)	99% A/sw/Jangsu/49/2010(H1N1)	99% A/sw/Jangsu/49/2010(H1N1)
**NA**	**NA**	99% A/Singapore/444W/2009(H1N1)	99% A/Viet Nam/13032011/2009(H1N1)	99% A/Viet Nam/13032011/2009(H1N1)
	**AA**	99% A/Wisconsin/629-D02329/2009(H1N1)	99% A/Japan/1070/2009(H1N1)	99% A/Florida/04/2009(H1N1)
**M**	**NA**	99% A/Singapore/GP293/2010(H1N1)	99% A/Singapore/GP1094/2009(H1N1)	99% A/Mexico City/INER18/2010(H1N1)
	**AA**	100% A/California/06/2009(H1N1)	100% A/Singapore/GP1094/2009(H1N1)	100% A/California/06/2009(H1N1)
**NS**	**NA**	99% A/sw/Indiana/SG1367/2010(H1N1)	99% A/Scotland/Fife_17690/2009(H1N1)	99% A/Singapore/ON2180/2009(H1N1)
	**AA**	99% A/sw/Tornitz/IDT14598/2012(H1N2)	99% A/sw/Tornitz/IDT14598/2012(H1N2)	99% A/Singapore/ON2180/2009(H1N1)

**gene**		**A/SW/ON/62/12/H1N1**	**A/SW/ON/68/12/H1N2**	**A/SW/ON/46/12/H1N1**
**PB2**	**NA**	99% A/Ontario/29801/2009(H1N1)	99% A/turkey/Ontario/31232/2005(H3N2)	99% A/sw/VIC/09-02767-01/2009(H1N1)
** **	**AA**	99% A/Ontario/29801/2009(H1N1)	99% A/turkey/Ohio/313053/2004(H3N2)	99% A/Barcelona/INS190/2009(H1N1)
**PB1**	**NA**	99% A/SW/SK/59-6/2009(H1N1)	99% A/sw/Manitoba/12707/2005(H3N2)	99% A/Oslo/INS404/2009(H1N1)
** **	**AA**	99% A/Canada-ON/RV2964/2009(H1N1)	99% A/sw/Manitoba/12707/2005(H3N2)	100% A/Mexico city/CIA9/2009(H1N1)
**PA**	**NA**	99% A/SW/SK/59-6/2009(H1N1)	99% A/SW/SK/59-6/2009(H1N1)	99% A/Viet Nam/13032036/2009(H1N1)
** **	**AA**	99% A/Terrassa/INS174/2009(H1N1)	99% A/Terrassa/INS174/2009(H1N1)	99% A/Bishkek/WRAIR0883N/2009(H1N1)
**HA**	**NA**	99% A/Singapore/420T/2009(H1N1)	99% A/Singapore/420T/2009(H1N1)	99% A/Ontario/309862/2009(H1N1)
** **	**AA**	99% A/Taiwan/8542/2009(H1N1)	99% A/Taiwan/8542/2009(H1N1)	99% A/Singapore/ON1576/2009(H1N1)
**NP**	**NA**	99% A/Mexico City/INERPHAC1/2009(H1N1)	99% A/sw/Manitoba/00446/2005(H3N2)	99% A/Ontario/315181/2009(H1N1)
** **	**AA**	99% A/sw/Jangsu/49/2010(H1N1)	99% A/sw/Mexico/SG1444/2011(H3N2)	100% A/sw/AR/SAGiles-31215/2009(H1N1)
**NA**	**NA**	99% A/Viet Nam/13032011/2009(H1N1)	99% A/turkey/Ontario/FAV-10/2011(H3N2)	99% A/Utah/51/2009(H1N1)
** **	**AA**	99% A/Mexico City/009/2009(H1N1)	99% A/sw/Indiana/A01260185/2013(H3N2)	99% A/Singapore/KK066/2010(H1N1)
**M**	**NA**	99% A/Singapore/TT37/2010(H1N1)	99% A/sw/Minnesota/00709/2005(H3N2)	99% A/Wisconsin/629-D01667/2009(H1N1)
** **	**AA**	100% A/California/06/2009(H1N1)	100% A/sw/MI/PU243/04 (H3N1)	100% A/Canada-ON/RV1527/2009(H1N1)
**NS**	**NA**	99% A/Singapore/ON2180/2009(H1N1)	97% A/sw/North Carolina/SG1170/2003(H1N1)	99% A/Singapore/ON2180/2009(H1N1)
** **	**AA**	99% A/Singapore/ON2180/2009(H1N1)	97% A/turkey/Ontario/FAV-10/2011(H3N2)	99% A/California/VRDL76/2009(H1N1)

^a^ Nucleotides

^b^ Amino acids

**Fig 4:**
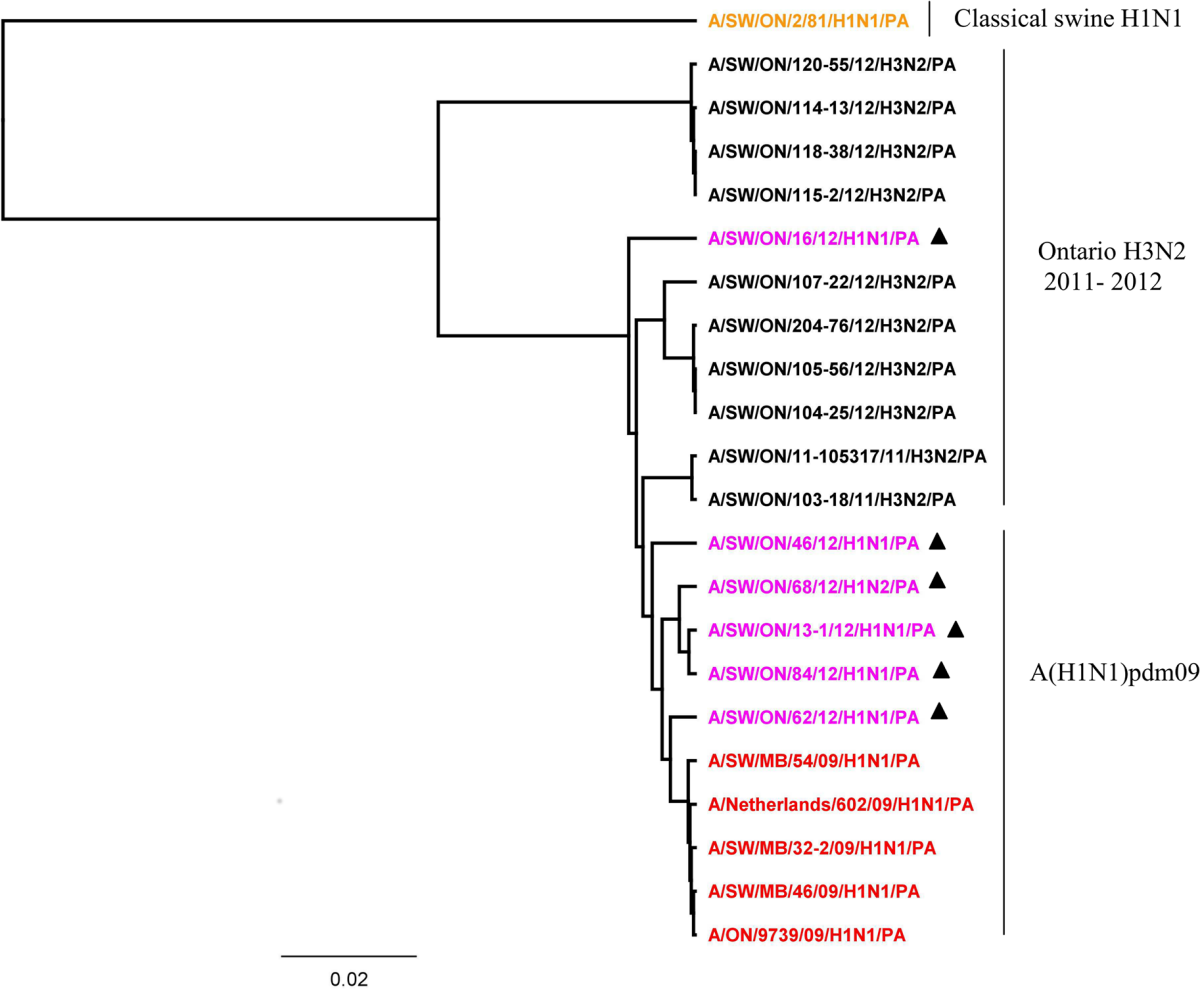
Phylogenetic analysis of the polymerase A (PA) gene of 5 Ontario H1N1 and 1 H1N2 viruses isolated from pigs in 2012 (purple with). Classical swine H1N1 (brown), Ontario H3N2 viruses isolated in 2011 and 2012 (black), and pandemic A(H1N1)pdm09 (red) have been included in analysis.

**Fig 5:**
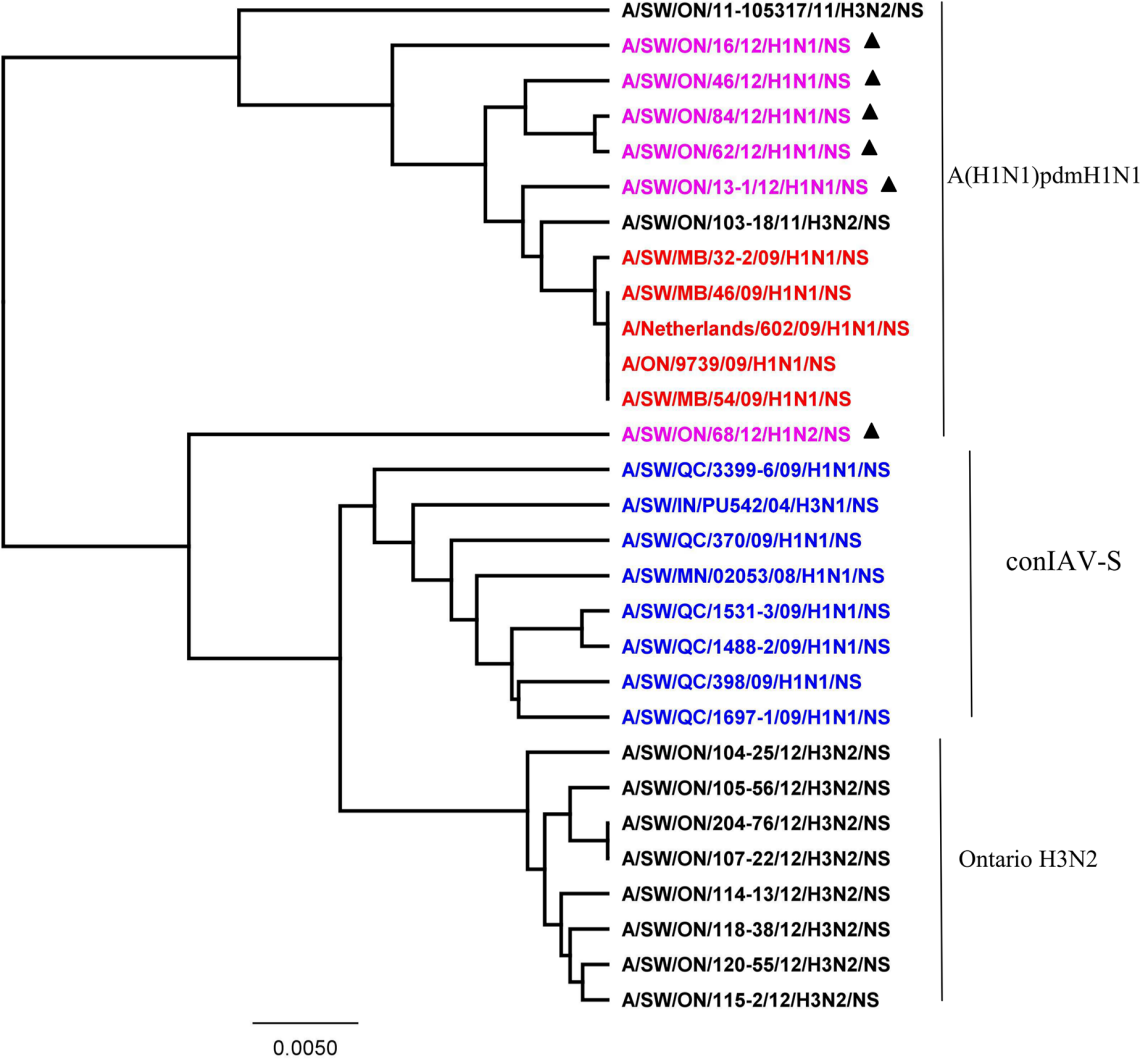
Phylogenetic analysis of nonstructural (NS) gene segments of 5 Ontario H1N1 and 1 Ontario H1N2 viruses isolated from pigs in 2012 (purple with ▲). Analysis includes contemporary influenza A viruses from swine (conIAV-S) which contain HA and NA genes of classical H1N1 in combination with the North American triple-reassortant internal gene (TRIG) cassette (blue), A(H1N1)pdm09 (red), and 2011–2012 Ontario H3N2 viruses (black).

More detailed analysis of the 2012 H1N2 virus isolated from Ontario pigs revealed that the HA, PA, and NS genes originated from A(H1N1)pdm09 and the NA, M, PB1, PB2, and NP genes originated from trH3N2 virus. The HA (Fig 1), PA (Fig 4), and NS (Fig 5) genes clustered with previously described A(H1N1)pdm09 viruses isolated from swine and humans. The HA, PA, and NS pairwise nucleotide identities between Ontario H1N2 and their counterparts of [A/ON/35273/2009/(H1N1)] and [A/Netherlands/602/2009/(H1N1)] were 99.1%, 99.1%, and 96.5% for HA, PA, and NS genes, respectively. As the NA and remaining internal genes (M, PB1, PB2, and NP) were genetically related to trH3N2 virus, their nucleotide sequence identities were compared to their respective 2012 Ontario H3N2 counterparts, and varied between 98.1% and 99.2%, 99.5% and 99.7%, 97.2% and 98%, 97.8%, and 97.9% and 98.7% for NA, M, PB1, PB2, and NP genes, respectively.

The percentage of genetic relatedness of the Ontario H1N2 isolate to other published influenza viruses in the NCBI database is presented in the Table 1. Based on the top matches for each gene sequence this 2012 H1N2 virus was derived from reassortment between A(H1N1)pdm09 and trH3N2 viruses.

### Resistance-associated mutations

**Fig 6:**
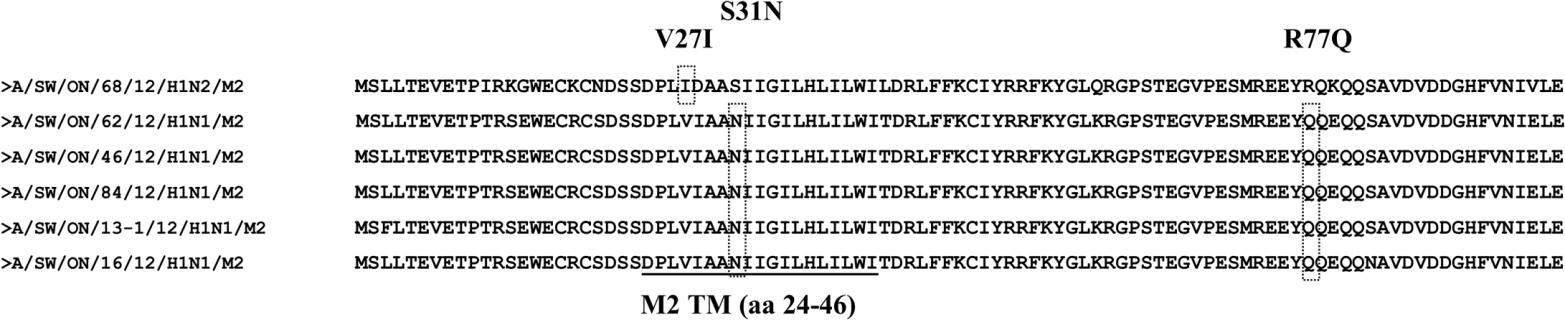
Potential resistance of 5 Ontario H1N1 and 1 H1N2 isolate to the M2 blocker amantadine. Alignment analysis of M2 sequences of Ontario H1N1 isolates showing amino acid substitution S31N. The M2 transmembrane is underlined.

The mutation responsible for resistance to adamantanes is an aa change from Ser to Asn at residue 31 (S31N) positioned in the transmembrane domain of the M2 protein. Sequence alignment analysis showed that the 5 Ontario H1N1 isolates do have the S31N mutation in the M2 protein, whereas the Ontario H1N2 isolate does not have such a mutation. Additional mutations, such as V27I and R77Q, have also been traced. The R77Q was found in the 5 Ontario H1N1 isolates, but not in the Ontario H1N2 isolate, which contained a V27I mutation (Fig 6). The V27I was absent from the Ontario H1N1 isolates (Fig 6). The H275Y, which is the NA mutation most frequently associated with oseltamivir resistance in the N1 subtype, was not detected in any of the Ontario H1N1 viruses. The NA mutations associated with oseltamivir resistance in the N2 subtype, such as E119V, R292K, and N294S, were not detected in the NA protein of the Ontario H1N2 virus. Additionally, the NA proteins of the 5 Ontario H1N1 isolates were compared to human A(H1N1)pdm09 isolate from Mexico [A/Mexico/InDRE4487/09]. Substitutions relative to the [A/Mexico/InDRE4487/09] are shown in the Table 2.

**Table 2:** Amino acid sequence alignment of the NA protein of 2012 Ontario H1N1 influenza A viruses from pigs.

Amino acids^a^																
Virus	30	72	82	95	106	111	117	221	232	248	309	313	382	388	449	452
A/Mexico/InDRE4487/09/NA	I	T	S	S	V	K	I	N	A	N	N	Q	G	I	N	T
A/SW/ON/13-1/12/H1N1/NA					I				V		D	K			H	
A/SW/ON/16/12/H1N1/NA		I			I	R		K		D					H	
A/SW/ON/84/12/H1N1/NA			P		I						D	K				I
A/SW/ON/62/12/H1N1/NA					I						D	K		T		I
A/SW/ON/46/12/H1N1/NA	T		A	R	I		M			D			E			

^a^Amino acid sequences of the 2012 Ontario H1N1 and H1N2 influenza A viruses from pigs were compared with A/Mexico/InDRE4487/09. Substitutions relative to the A/Mexico/InDRE4487/09 are shown. Empty cells indicate there were no substitutions.

### Signature residues

**Fig 7:**
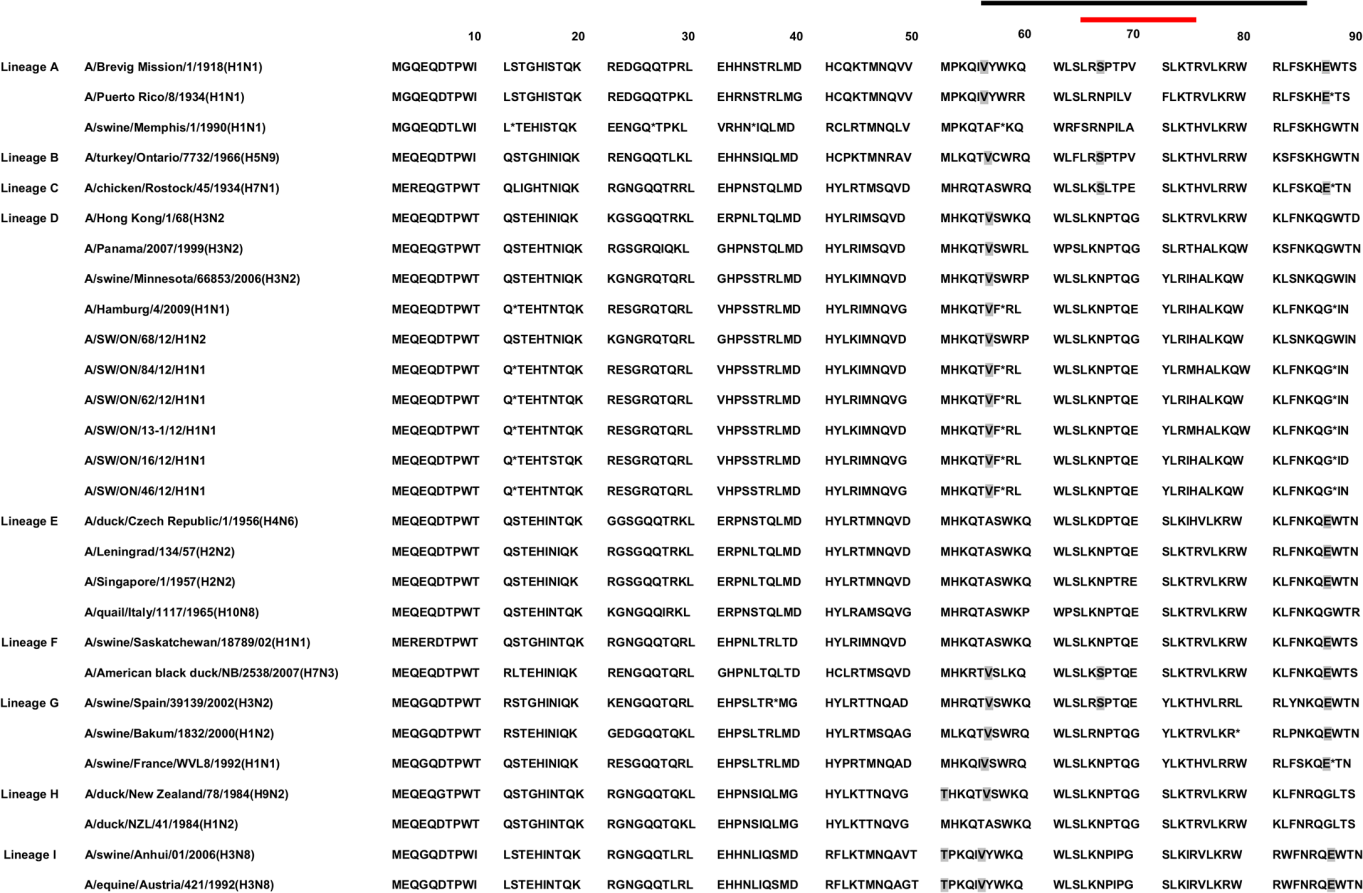
Presentation of polymerase B1-F2 (PB1-F2) variants of mammalian and avian influenza A viruses. Nine PB1 lineages (A to I) have been described and presented. Colored lines at the top represent amino acids of the predicted helical region (black) and the putative mitochondrial targeting sequence (red). Amino acids that are considered to enhance viral pathogenesis are marked in gray (T51, V56, N66S, E87). The first stop codon has been shown by asterisk and following stop codons are indicated by asterisk.

**Fig 8:**
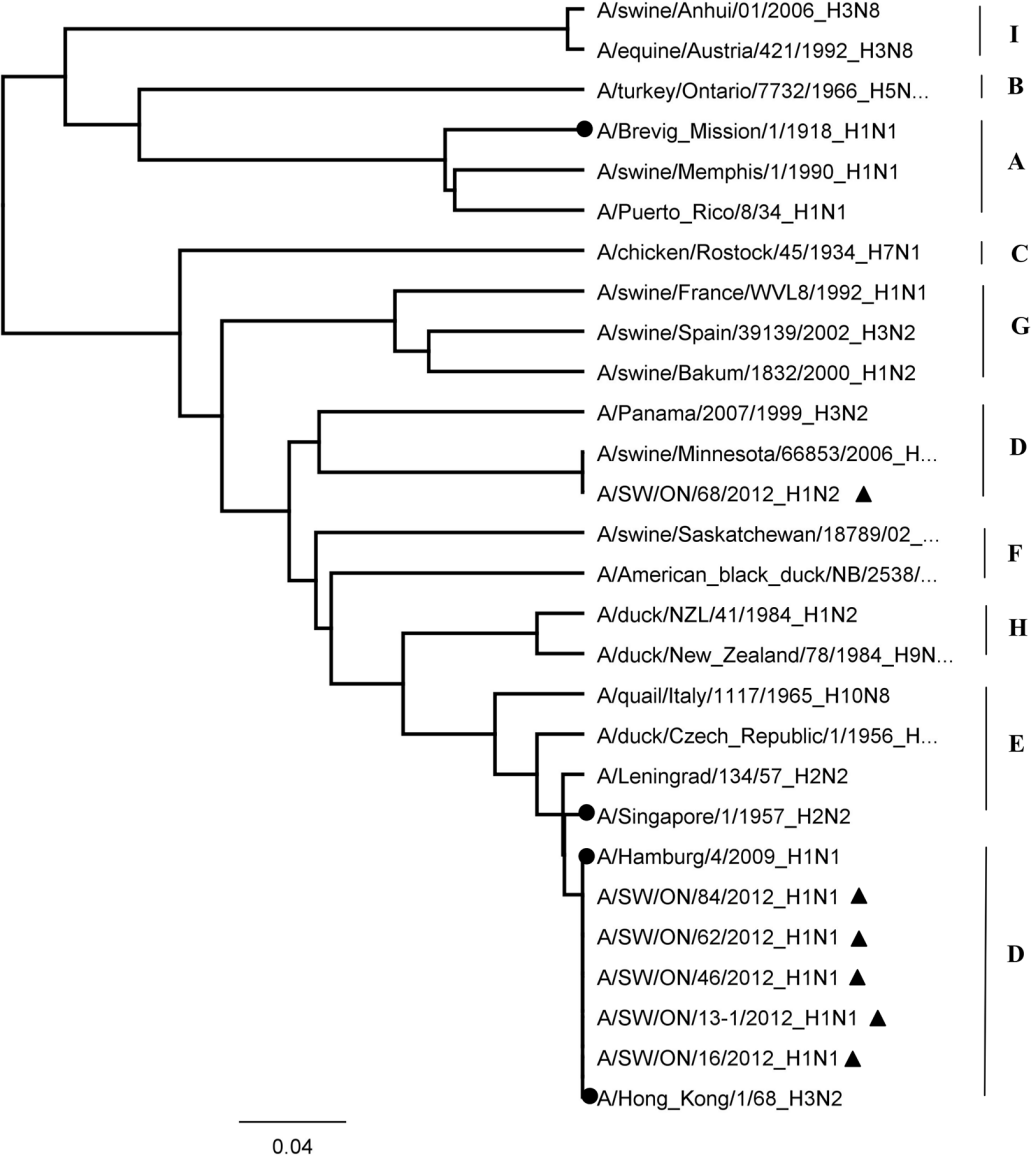
Phylogenetic analysis of 28 polymerase B1-F2 (PB1-F2) sequences. Representatives of the nine PB1 lineages (A to I) were selected. The pandemic strains of 1918 (H1N1), 1957 (H2N2), 1968 (H3N2) and H1N1pdm09 are marked with (●). The H1N1 and H1N2 viruses isolated from pigs in 2012 are marked with (▲).

The PB1 non-structural protein of influenza A virus, encoded by segment 2 of the viral genome, is an essential component of the viral polymerase. The mRNA transcribed from segment 2 encodes 3 proteins: PB1, PB1-F2 encoded by the +1 alternate open reading frame (ORF) of segment 2, and PB1-N40. All 5 Ontario H1N1 viruses harbored a truncated form of PB1-F2 containing 11 aa, lacking the mitochondrial targeting sequence (MTS) positioned in the C-terminal region. The Ontario H1N2 virus harbored a complete 90 aa PB1-F2 fragment. More detailed protein sequence analysis of all 6 Ontario PB1-F2 proteins revealed the absence of the N66S mutation previously reported in the H1N1 1918 PB1-F2 virus, associated with an increase in virulence in a mouse model (Fig 7). Additional aa associated with enhanced pathogenicity in avian influenza virus [24], such as T51, V56, and E87, have also been explored. As can be seen from Fig 7, the V56 has been determined in all 6 Ontario PB1-F2 proteins; however, T51 and E87 were absent. Phylogenetic analysis of 28 PB1-F2 aa sequences showed that Ontario H1N1 and H1N2 viruses belong to lineage D (Fig 8).

### HA1 amino acid analysis

**Fig 9:**
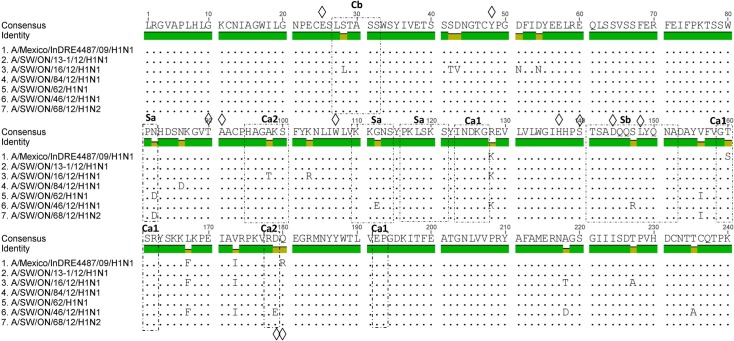
Predicted HA1 proteins of the 2012 Ontario H1 viruses. The six 2012 Ontario H1 predicted HA1 proteins were aligned and compared to that of 2009 pandemic H1N1 (A/Mexico/InDRE4487/2009) as reference. Amino acid differences are visible in the antigenic sites Cb (1), Sa (3), Ca2 (2), Ca1 (6), Sb (1). Receptor-binding pocket residures are indicated by a diamond.

**Fig 10:**
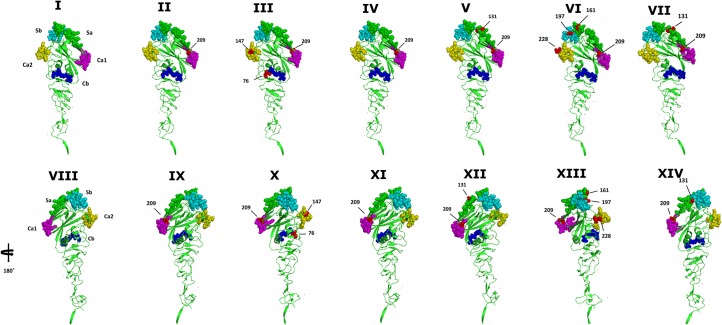
Positions of the amino acid alterations at major antigenic sites of the HA1 molecule in the H3 crystal structure. The HA monomer in cartoon format with major antigenic sites: Sa (green), Sb (cyan), Ca1 (magenta), Ca2 (yello) and Cb (blue) shown in spheres (I). The positions of changed amino acids are shown in red for each of 6 Ontario 2012 viruses: II (A/SW/ON/13-1/12/H1N1), III (A/SW/ON/16/12/H1N1), IV(A/SW/ON/84/12/H1N1), V (A/SW/ON/68/12/H1N1, VI (A/SW/ON/46/12/H1N1, VII (A/SW/ON/62/12/H1N1). The view of panels I to VII is rotated 180°C along Y-axis and positions of changed amino acids are shown in red (VIII, IX, X, XI, XII, XIII, XIV).

To determine whether the aa changes occurred in any of the known antigenic sites Sa, Sb, Ca1, Ca2, Cb [25], we aligned the aa sequences of all 6 Ontario H1 predicted HA1 proteins and compared them to those of a human A(H1N1)pdm09 isolate from Mexico [A/Mexico/InDRE4487/09], apparently a swine-origin virus. The HA gene of all 6 Ontario H1 showed a high identity (96.9% to 99%) with the human A(H1N1)pdm09 from Mexico. When compared to the A(H1N1)pdm09 [A/Mexico/InDRE4487/09], each of the 5 Ontario H1N1 viruses had between 1 and 4 aa changes within 5 antigenic sites, Sa, Sb, Ca1, Ca2, and Cb. The A/SW/ON/16/2012/H1N1 had 3 aa changes within 3 antigenic sites Cb (1), Ca1 (1), and Ca2 (1). Both A/SW/ON/13-1/2012/H1N1 and A/SW/ON/84/2012/H1N1 had 1 aa change within the same antigenic site Ca1 (2). The A/SW/ON/62/2012/H1N1 had 2 aa changes within 2 antigenic sites, Sa (1) and Ca1 (1), and A/SW/ON/46/2012/H1N1 had 4 aa changes within 4 antigenic sites, Sa (1), Sb (1), Ca1 (1), and Ca2 (1). The 1 Ontario H1N2 virus had 2 aa changes within 2 antigenic sites, Sa (1) and Ca1 (1) (Fig 9). Additionally, the presence of 144D suggests a human Neu5Acα2-6Gal receptor binding preference [12]. Amino acid differences in the antigenic sites and residues of the receptor binding pocket are presented in Fig 9 and Fig 10. This structural modeling shown in Fig 10 helps to visualize these changes. Moreover, full HA aa sequences of all 6 Ontario H1 isolates were compared to the human isolate [A/Mexico/InDRE4487/09] and substitutions relative to the [A/Mexico/InDRE4487/09] are shown in Table 3.

**Table 3:** Amino acid sequence alignment of the HA1 and HA2 protein of 2012 Ontario H1N1 and H1N2 influenza A viruses from pigs.

	Amino acids^a^																																
	HA1																									HA2							
Isolate	**19**	**71**	**85**	**86**	**94**	**97**	**125**	**129**	**141**	**146**	**155**	**171**	**190**	**199**	**203**	**210**	**216**	**222**	**223**	**261**	**270**	**278**	**297**	**300**		**449**	**451**	**479**	**485**	**504**	**520**	**525**	**547**
A/Mexico/InDRE4487/09	V	S	S	D	D	D	N	N	A	K	G	K	S	V	S	F	I	D	R	A	T	T	P	I		V	S	V	D	K	V	V	I
A/SW/ON/13-1/H1N1												R			T	L	V		Q														
A/SW/ON/16/H1N1	I	L	L	V	N	N			T	R					T				Q	T	A		S	V				I	N	E			
A/SW/ON/84/H1N1								D				R			T	L	V		Q												A	I	
A/SW/ON/62/H1N1							D					R		I	T	L	V		Q														
A/SW/ON/68/H1N2							D					R		I	T	L	V		Q														
A/SW/ON/46/H1N1											E		R		T			E	Q	D		A				I	N						V

^a^Amino acid sequences of the 2012 Ontario H1N1 and H1N2 influenza A viruses from pigs were compared to the human isolate A/Mexico/InDRE4487/09. Substitutions relative to the A/Mexico/InDRE4487/09 are shown. Empty cells indicate there were no substitutions.

## Discussion

Here we describe genetic and predicted antigenic characterization of the 5 Ontario H1N1 and 1 H1N2 virus swine isolates isolated in 2012. On the basis of these results and previously published material [21], it can be concluded that pandemic H1N1 IAV-S, together with H1N2 and at least three different groups of H3N2 viruses were circulating in Ontario during 2011 and 2012. In the 5 Ontario H1N1 viruses, all eight genes were genetically related to A(H1N1)pdm09. In the H1N2 virus, the HA, PA, and NS genes were closely related to A(H1N1)pdm09 and the NA, M, PB1, PB2, and NP genes originating from trH3N2 virus. Structurally similar viruses had already been observed in Canada since A(H1N1)pdm09 emerged in 2009 [23]. Interestingly, classical swine H1N1 could not be detected in this study. Classical strains of H1N1 may have been better detected if virus isolation in eggs had been used in addition to virus isolation in MDCK cells. More testing is needed to confirm whether the classical swine H1N1 is still circulating in this population.

The HA1 antigenic site analysis has been performed employing the human A(H1N1)pdm09 virus from Mexico [A/Mexico/InDRE4487/09] as the reference. This previously unrecognized H1N1 swine-origin subtype, containing a gene combination of North American and Eurasian IAV-S, was isolated from humans in the U.S. and Mexico. The virus spread swiftly and globally as the first pandemic of the 21th century. Soon after the first isolation of virus from humans in the U.S. and Mexico, Howden et al. [26] and Berhane et al. [27] reported human-to-animal transmission in Canada. Canadian virus isolates from swine were phylogenetically similar to A(H1N1)pdm09 virus [26,28] and have been often detected in the Canadian swine population [23]. The percentage of aa identity of HA1 between the [A/Mexico/InDRE4487/09] and the 6 Ontario H1 subtype viruses ranged from 95.4% to 97.9%. The HA1 aa sequences of all 6 Ontario IAV-S showed aa changes in an antigenically important region. We detected 1 to 4 aa changes within 5 antigenic sites: Cb (1) S28L, Sa (3) N82D and G112E, Sb (1) S147R, Ca1 (6) S160T, and Ca2 (2) A98T and D179E. According to Wood et al. [29] and Kodihalli et al. [30] 1 to 3 aa changes in the HA1 of H1N1 or H3N2 viruses are sufficient to reduce cross reactivity and efficacy of inactivated vaccine. Therefore, observed aa changes in the antigenic sites of the 6 Ontario isolates suggest virus fitness probably due to virus antibody escape that consequently could have an effect on antibody recognition. This, however, should be investigated in further antigenic studies.

The PB1-F2 is a non-structural, short (87–101 aa) protein of influenza A virus, reported in 2001 by Chen et al. [31]. The Ontario H1N1 viruses have an 11 aa long PB1-F2 protein. The PB1-F2 protein of the Ontario H1N1 viruses contains a C129A point mutation in the PB1 gene. This mutation leads to the formation of stop codon at the 12^th^ aa position, with additional stop codons placed at positions 58 and 88. A similar finding was reported by Smith et al. [32] regarding the H1N1 2009 pandemic virus. The Ontario H1N2 virus does not have a C129A mutation, and therefore the Ontario H1N2 PB1-F2 protein is 90 aa long. An N66S (arginine to serine) mutation in the C-terminal region of the PB1-F2 has been correlated with apoptotic function of the protein. This N66S substitution was reported first in the 1918 pandemic H1N1 virus and the highly pathogenic H5N1 virus of the 1997 Hong Kong outbreak [33]. Interestingly, N66S substitution is absent in all Ontario isolates, and H1N1 isolates contain a truncated PB1-F2. However, those isolates can acquire the mutation either through error-prone RNA polymerase or through ressortment with a virus that contains the N66S mutation.

M2 is a 97-residue long membrane protein, with 23 aa at the N-terminus, 19 residues representing the transmembrane domain (M2TM), and 55 residues residing in the cytoplasmic tail [34]. M2TM is the known target of the anti-influenza drugs amantadine and rimantadine. These two antivirals have been the first line of defense for some time in order to reserve the neuraminidase inhibitors in the event of an epidemic. Over the past decade, approximately 97% of influenza strains have developed resistance to amantadine and rimantadine [34]. In agreement with previously published findings, the Ontario H1N1 and H1N2 viruses also contained resistance-associated mutations to adamantanes. S31N has been detected in all 5 Ontario H1N1 viruses, but was absent from the Ontario H1N2 virus. However, additional mutations, such as V27I, have been found in H1N2 virus and R77Q has been found in all 5 H1N1 viruses.

In conclusion, the results of this study suggest that the H1N1 of the 2009 pandemic lineage was the dominant genotype of H1N1 in Ontario pigs in 2012, a situation that resembled findings from other geographical areas [35,36,37]. The H1N1 viruses detected in this study contained gene segments with high similarity to viruses that circulated in human populations in 2009. Moreover, sequenced viruses exhibited 1 to 4 aa changes located in the most variable parts of the HA molecule, i.e., the HA1 region. Amino acid changes occurring in antigenic sites may have significant effects on antibody recognition end efficacy of inactivated vaccines. This also suggests the possibility of important antigenic differences, which should be further confirmed in antigenic studies. In addition, a reassortant H1N2 virus containing HA, PA, and NS genes from 2009(H1N1)pdm and NA, M, PB1, PB2, and NP genes from trH3N2, was also detected, again in line with similar observations from other geographical areas [38,39]. According to aa sequence analysis of the M2 protein, Ontario H1N1 and H1N2 viruses can be expected to offer resistance to amantadine and rimantadine due to different mutations, but not to neuraminidase inhibitors. Additionally, absence of the N66S substitution in 2012 Ontario swine isolates suggests that these viruses might have low virulence, a finding that should be further investigated in pathogenicity studies.
